# Outlook on PI3K/AKT/mTOR inhibition in acute leukemia

**DOI:** 10.1186/s40591-015-0040-8

**Published:** 2015-03-20

**Authors:** Lars Fransecky, Liliana H Mochmann, Claudia D Baldus

**Affiliations:** Department of Hematology and Oncology, Charité University Hospital Berlin, Campus Benjamin Franklin, Berlin, Germany

**Keywords:** Acute leukemia, PI3K, AKT, mTOR, Targeted therapy

## Abstract

Technological advances allowing high throughput analyses across numerous cancer tissues have allowed much progress in understanding complex cellular signaling. In the future, the genetic landscape in cancer may have more clinical relevance than diagnosis based on tumor origin. This progress has emphasized PI3K/AKT/mTOR, among others, as a central signaling center of cancer development due to its governing control in cellular growth, survival, and metabolism. The discovery of high frequencies of mutations in the PI3K/AKT/mTOR pathway in different cancer entities has sparked interest to inhibit elements of this pathway. In acute leukemia pharmacological interruption has yet to achieve desirable efficacy as targetable downstream mutations in PI3K/AKT/mTOR are absent. Nevertheless, mutations in membrane-associated genes upstream of PI3K/AKT/mTOR are frequent in acute leukemia and are associated with aberrant activation of PI3K/AKT/mTOR thus providing a good rationale for further exploration. This review attempts to summarize key findings leading to aberrant activation and to reflect on both promises and challenges of targeting PI3K/AKT/mTOR in acute leukemia. Our emphasis lies on the insights gained through high-throughput data acquisition that open up new avenues for identifying specific subgroups of acute leukemia as ideal candidates for PI3K/AKT/mTOR targeted therapy.

## Review

### Introduction

Interest into targeting the phosphoinositide 3-kinase, AKT, and mammalian target of rapamycin (PI3K/AKT/mTOR) signaling network in cancer has increased by the recent disclosure that PIK3CA of the PI3K pathway is the second most frequently mutated gene in cancer [1]. Overall, a number of elements of the PI3K/AKT/mTOR pathway are frequently mutated in cancer, thus stimulating strong interest for PI3K-specific drug development. Considerable efforts have led to numerous clinical trials of PI3K/AKT/mTOR inhibition in cancer, including in acute leukemia. As a result, PI3K/AKT/mTOR-directed therapies are promoted as the standard of care in some cancer (e.g. renal cell carcinoma [2]), but not in acute leukemia. This shortfall may be due to the lack of targetable mutations, as all major components of PI3K/AKT/mTOR have a lower frequency of mutations or copy number variations (CNV) in acute leukemia than in other cancer types [1] (see Table 1). On the other hand, genetic alterations in receptor tyrosine kinases (RTKs) constitute one of the major sources of aberrant upstream activation of PI3K/AKT/mTOR (see Table 1). Drugs designed to target mutant RTKs have shown clinical efficacy and enhanced our understanding of the mechanisms of cell signaling [3], but are often limited by drug resistance.

**Table 1:** **A comparison of the frequency of mutations and copy number variations** (**CNV**) **in the components of the PI3K**/**AKT**/**mTOR pathway in AML**, **T**-**ALL**, **and B**-**ALL**

**Mutations**						
	**Gene**	**Description**	**AML SNP**	**AML CNV**	**T** **-ALL SNP**	**B** **-ALL SNP**
PI3K/AKT/mTOR	PIK3CA	phosphatidylinositol-4,5-bisphosphate 3-kinase, catalytic subunit alpha	0% (228)	0.5% (188) loss	0% (20)	0% (9)
	PIK3CB	phosphatidylinositol-4,5-bisphosphate 3-kinase, catalytic subunit beta	0% (228)	0.5% (188) loss	0% (20)	0% (9)
	PIK3CD	phosphatidylinositol-4,5-bisphosphate 3-kinase, catalytic subunit delta	0% (228)	2.71% (188) gain, 0.5% (188) loss	0% (28)	0% (9)
	PIK3CG	phosphatidylinositol-4,5-bisphosphate 3-kinase, catalytic subunit gamma	0% (228)	9% (188) loss	0% (28)	0% (9)
	PTEN	phosphatase and tensin homolog	0.48% (417)	1.1% (188) gain, 0.5% (188) loss	15.66% (862)	0% (81)
	AKT1	v-akt murine thymoma viral oncogene homolog 1	0% (571)	1.1%, (188) gain, 2.7% (188) loss	1.9% (203)	0% (76)
	AKT2	v-akt murine thymoma viral oncogene homolog 2	0% (228)	4.8% (188) gain	0% (22)	0% (9)
	AKT3	v-akt murine thymoma viral oncogene homolog 3	0% (228)	2.1% (188) gain, 0.5% (188) loss	0% (20)	0% (9)
	mTOR	mechanistic target of rapamycin (serine/threonine kinase)	0.44% (228)	2.71% (188) gain, 0.5% (188) loss	0% (20)	0% (9)
	PI3KR1	phosphoinositide-3-kinase, regulatory subunit 1 (alpha)	0% (328)	0.5% (188) gain, 2.71% (188) loss	0.56% (178)	0% (58)
Receptors	TLR4	toll-like receptor 4	0.44% (228)	3.7% (188) gain, 0.5% (188) loss	0% (20)	0% (9)
	NRAS	neuroblastoma ras viral oncogene	10.69% (4351)	1.1% (188) gain, 0.5% (188) loss	10.36% (502)	0% (818)
	KRAS	phosphatase and tensin homolog	4.1% (2339)	1.6% (188) loss	1.75% (456)	10,36% (772)
	EGFR	epidermal growth factor receptor	0.44% (229)	6.9% (188) loss	13.79% (29)	0% (9)
	FLT3	fms-related tyrosine kinase 3	23.75% (62135)	1.6% (188) gain, 1.1% (188) loss	4,59% (740)	4,04% (792)
	EPHA3	EPH receptor A3	0.44% (229)	2.7% (188) loss	0% (20)	0% (9)
	ERBB4	v-erb-b2 avian erythroblastic leukemia viral oncogene homolog 4	0% (228)	0.5% (188) gain	0% (20)	0% (9)
	PDGFRA	platelet-derived growth factor receptor, alpha polypeptide	1.02% (394)	2.1% (188) gain	11.54% (26)	0% (9)
	EPHB6	EPH receptor B6	0% (228)	0.5% (188) gain, 10.6% (188) loss	0% (20)	0% (9)
	FGFR2	fibroblast growth factor receptor 2	0% (228)	1.1% (188) gain	8.33% (24)	0% (9)
	KIT	v-kit Hardy-Zuckerman 4 feline sarcoma viral oncogene homolog	8.82% (5895)	2.1% (188) gain	0% (1)	0% (10)
	FGFR3	fibroblast growth factor receptor 3	0% (228)	1.6% (188) gain, 2.1% (188) loss	23.33% (30)	0% (9)

Frequency and cohort size are indicated. Mutational frequencies and CNVs were obtained from COSMIC repository [48] v69.

Listed are the major PI3K/AKT/mTOR components including gene symbols, description of the genes, and frequency of mutation in each disease and CNV in AML.

We describe herein that PI3K/AKT/mTOR is a central circuit within the pathogenesis of acute leukemia and summarize both molecular and clinical findings that may aid in developing ways to overcome the oncogenic potential of PI3K/AKT/mTOR.

### Treatment of acute leukemia

Acute leukemia is a heterogeneous clonal disease with malignant cells characterized by both growth advantage and block in differentiation. Classified by the cell of origin, acute myeloid leukemia (AML) separates from acute lymphoblastic leukemia of B- or T-cell origin (B-precursor ALL, T-ALL). An estimated 140,000 people worldwide are diagnosed with acute leukemia each year [4]. In general, treatment consists of induction chemotherapy, capable of inducing remission in approximately 75% of all cases [5,6]. Consecutive consolidation treatment, too, pillars on chemotherapy. Overall survival across all risk groups is achieved in only 50% in ALL [7] and with 35% slightly less in AML [8] and it is generally accepted that chemotherapy alone will only marginally be able to increase outcome in the future.

Enhanced understanding of leukemogenesis at the molecular level has allowed identifying subgroups with higher probability to respond to targeted therapy. In B-ALL, for instance, the presence of translocation t(9;22) (q34;q11) [9] leads to a fusion gene called BCR-ABL in 15-30% of adult B-precursor ALL patients [10]. The fusion product of this protein, a constitutively activated tyrosine kinase, can be specifically inhibited by a range of tyrosine kinase inhibitors (e.g. imatinib) and both response rates and overall survival have substantially increased in this very high risk B-precursor ALL [11]. Moreover, in AML, the translocation t(15;17)(q22;q12) produces a fusion protein called PML-RARA [12], which is pathognomonic for acute promyelocytic leukemia (APL). Chemotherapy-free treatment with arsenic trioxide (ATO) and retinoic acid (ATRA) induced complete remission in all of 71 patients with two year overall survival reported at 97% in low/intermediate risk APL [13].

While major improvements through targeted therapies serve as proof-of-principle, it is undeniable that genetically driven therapy is still not available for the majority of patients. In unselected cohorts, targeted therapies, in particular as monotherapy, have generally produced disappointing results regarding response rates as well as duration likely due to the fact that common molecular targets shared in all cases of a disease are a rarity. Therefore, to bring targeted therapy to its full clinical use, the principle of stratification is essential to predict subgroups of patients with sensitivity to a given treatment. The need of molecular understanding of the target as well as downstream effects of target inhibition is a prerequisite for successful application of signal transduction inhibition (STI) such as PI3K/AKT/mTOR.

### Key regulators of PI3K/AKT/mTOR in hematopoiesis

PI3K/AKT/mTOR signaling controls proliferation, differentiation, and survival of hematopoietic cells. In this section, we aim to briefly describe the key genes of the PI3K/AKT/mTOR pathway with significant impact in normal hematopoiesis and refer to further in-depth molecular reviews [14,15].

Under normal conditions, PI3K activation is initiated through extracellular binding of ligands (e.g. EGF, HER2, KIT ligand, PDGF, MET), which in turn triggers the activation of corresponding receptor tyrosine kinases (RTK). The plethora of receptors may include insulin receptor (IR), Fms-like tyrosine kinase 3 (*FLT3*), c-KIT, epidermal growth factor receptor (*EGFR*), platelet derived growth factor receptor alpha (*PDGFRa*), fibroblast growth factor receptor (*FGFR*), colony-stimulating growth factor I (*CSF*-*I*) or insulin-like growth factor I (*IGF*-*I*).

To date, three distinct classes of PI3Ks have been defined of which only class I is discussed in this review. Class I PI3K is comprised of five different regulatory subunits (p85α, p85β, p55α, p55γ, or p50α) and four catalytic subunits (p110α, p110β, p110γ, and p110δ. While p110α and p110β are ubiquitously expressed, the expression of the catalytic isoforms p110δ or p110γ are restricted to white blood cells [16]. The regulatory and catalytic subunits are constitutively associated and upon activation, the p85/p110 heterodimer undergoes a conformational change releasing the catalytic activity of p110.

PI3K activation is described to be initiated through several distinct mechanisms, beginning with RTK dimerization and autophosphorylation at tyrosine residues to allow the interaction with src homology domain containing proteins. One possible mechanism includes direct binding of the regulatory subunit p85 to RTK followed by the activation of the catalytic p110 subunit of PI3K. Another possibility is for the adaptor protein growth factor receptor-bound protein 2 (GRB2) to bind RTK directly, which in turn utilizes the scaffolding protein GAB (GRB2-associated binding protein) to bind p85 regulatory subunit. Alternatively, GRB2 may activate p110 without the p85 regulatory subunit involving RAS.

Recruitment of PI3K leads to the conversion of phophatidylinositol-4,5-bisphosphate (PIP2) to phosphatidylinositol-3,4,5-trisphosphate (PIP3). PIP3 recruits AKT and phosphoinositide-dependent kinase 1 (PDK1) to the plasma membrane resulting in AKT phosphorylation by PDK1 at Thr308. For full activation, AKT is also phosphorylated by mTORC2 at Ser473. The subsequent steps from this point onwards are numerous, as studies have now uncovered more than 100 AKT substrates. At this branching point, the network has significant and crucial access to other signaling pathways and some branches include GSK3, FOXO, BAD, IKK/®, eNOS or p21^Cip1^.

With particular relevance to both normal and malignant hematopoiesis, AKT phosphorylation induces the phosphorylation of TSC2, which in turn acts as a GTPase activating protein (GAP) for the GTPase RAS homologue enriched in brain (Rheb). Because AKT inhibits TSC2 through phosphorylation, it permits mTORC1 activation by Rheb. Activated mTORC1 in turn regulates S6K phosphorylation/activation for protein production and phosphorylation/inactivation of 4EBP. Besides its activation of protein production, mTORC1 phosphorylation of S6K is known to induce phosphorylation of other adaptor proteins such as insulin receptor substrate 1 (IRS-1) to attenuate growth factor signaling through PI3K/AKT/mTOR [17]. The inactivation of 4EBP will cause the repression of eIF4E-dependent initiation of (cap-dependent) translation. Thus, these complexes place mTORC1 at the center of protein synthesis (reviewed in [18]).

Regulation of PI3K activity is negatively controlled by de-phosphatases, such as *PTEN* and Src homology domain containing inositol phosphatases (SHP1 and SHP2). PIP3 hydrolyzed by PTEN generates PIP2, bringing PI3K signaling back to steady state [15]. The functional importance of *PTEN* is emphasized by the fact, that it is the third most mutated gene in human cancer [1].

### PI3K/AKT/mTOR alterations in acute leukemia

#### Acute myeloid leukemia (AML)

In 1998, successful cloning of PI3K consisting of a mutant p65 regulatory subunit led to malignant cellular progression by constitutive activation of the catalytic subunit [19]. Both mTOR and AKT activation have long been considered downstream effects of PI3K activation in AML (reviewed in [18]. Direct evidence of constitutive PI3K activation in AML was initially described in 2004, when expression of the p85α subunit of PI3K was detected in nearly all AML samples and of 40 AML patients, 21 cases exhibited increased PI3K activity. Moreover, PI3K expression correlated with proliferation in AML blasts [20]. Accordingly, treatment of primary AML blasts with LY294002, an unselective inhibitor of PI3K and all the PI3K-related kinase (PIKK) family, resulted in the induction of apoptosis *in vitro* and impaired engraftment in NOD/SCID mice *in vivo* [21]. Additional evidence implicating PI3K in the pathogenesis of AML surfaced, when knock-out of PI3Kα (encoded by Pik3ca) in a murine model with overactive KRAS, (i.e. KRASG12D) improved survival [22].

The catalytic subunits of class I PI3K molecules (i.e. p110α, p110β, p110γ and p110δ) are responsible for AKT activation. In AML, unlike all other isoforms, p110δ is consistently expressed at high levels and the p110δ-specific inhibitor IC87114 was capable of suppressing AKT activation to the same degree as unspecific LY294002 [23,24]. Moreover, IC87114 impaired proliferation of AML blast while sparing normal hematopoietic stem cells (HSCs).

Activation of AKT is mediated by phosphorylation at Thr308 by PDK1 and Ser473 by mTORC2. In AML, constitutive activation at Thr308 and Ser473 was detected in 50 - 72% of patients respectively [25,26]. While Gallay and colleagues reported that AKT phosphorylation at Thr308 was associated to shorter overall survival (OS) [27], the prognostic impact of AKT activation on Ser473 in AML was not clear without ambiguity. In a study of 61 patients who were not all eligible for intensive chemotherapy, Min et al. reported that AKT phosphorylation on Ser473 was associated to an inferior OS [26]. Kornblau et al. reported similar results in a cohort of 188 patients including about a third of patients with secondary AML [28]. In contrast, phosphorylation at Ser473 represented an independent favorable prognostic factor in a cohort of 92 patients [25]. Of note, AKT phosphorylation was detected to a lesser degree (i.e. 50%) in the latter trial, which, along with differences in patient cohorts may explain the differences in the results.

Exploring possible mechanisms of constitutive AKT activation in AML, the presence of *FLT3*-ITD, the most common mutation in AML, was identified as a source of dysregulation [29]. Downstream effects included inactivation of *FOXO3a* through phosphorylation and restoration of *FOXO3a* was capable of reversing FLT3-ITD^+^/AKT mediated growth advantages [29]. Another mechanism for constitutive AKT activation in AML was autocrine IGF-1/IGF-1R signaling in AML as inhibition of IGF-1R resulted in attenuating AKT activation in 70% of PI3K activated samples [30].

One important downstream target of AKT is mTORC1, which was also reported to be activated in AML. Phosphorylation of downstream targets such as p70S6, S6RP and 4EBP1 was detected in nearly all AML cases [31,32]. Importantly, pS6RP could not only be mediated through PI3K- or mTOR inhibitors but also through MAPK inhibitors. Thus, PI3K-independent activation of mTORC1 may prove to be of critical importance for clinical applications. Also, mTOR inhibition resulted in anti-leukemic activity *in vitro* and *in vivo* when combined with chemotherapy [21,33].

*PTEN*, the third most frequently mutated gene in human cancer, is very rarely mutated in AML (Table 1) [1,34]. Aberrant *PTEN* transcripts have been detected in a subset of AML patients [35] and phosphorylation was associated with increased *AKT* signaling and poor outcome [36].

One major source of PI3K/AKT/mTOR dysregulation stems from mutations in membrane bound proteins, such as RTKs (e.g. c-KIT or FLT3-ITD) or GTPases (e.g. KRAS, NRAS). Mutations in these proteins were observed in 55% of AML cases [1,34] and are associated to PI3K/AKT/mTOR activation [37]. Brandts, 2005 [29] Therefore, synergistic effects of combinational therapy targeting both RTK and PI3K/AKT/mTOR might be exploited in future clinical trials.

### B precursor lymphoblastic leukemia-ALL (B-ALL)

Like in AML, PI3K/AKT/mTOR activation is frequently found in B-ALL. A prominent model of PI3K activation in B-ALL comes from its activation through the BCR-ABL oncogene in Ph^+^ B-ALL. In 1995, p210 bcr-abl was shown to interact with the p85 subunit of PI3K thereby increasing PI3K activity substantially in a model for chronic myelogenous leukemia [38]. However, it remained unclear to which extent these observations could be transferred to B-ALL. More mechanistic insights were provided through a murine model of Ph^+^ B-ALL with ablation of *Pik3r1* and *Pik3r2*. Without these genes coding for PI3K regulatory isoforms, p190 bcr-abl mediated transformation was impaired [39]. Among the isoforms of the catalytic subunit of class I PI3K, p110δ is the most promising target for inhibition in B-ALL given the restricted expression of the p110δ isoform on leukocytes [16] and its important role in B cell signaling [40].

AKT activation is significantly higher in B-ALL compared to healthy bone marrow [41]. In pediatric patients with pre-B-ALL, pAKT correlated with poor response to chemotherapy and overexpression of pAKT *in vitro* sufficed to reverse the induction of apoptosis by standard anti-leukemic drugs, such as dexamethasone, vincristine or adriamycin. Moreover, in a retrospective study, pAKT was associated to poor overall survival [42].

Upon mTOR inhibition with rapamycin, B-ALL blasts demonstrated growth inhibition *in vitro* and *in vivo*. The fact, that Eμ-ret transgenic mice, a model for pre-B-ALL, displayed a survival benefit under treatment with rapamycin was commonly considered an indication of aberrant mTOR inhibition [43]. Ph-like B-ALL is a high risk subtype with frequent alterations in IKZF1, CRLF2 and JAK and a distinct gene expression profile resembling that of Ph^+^ B-ALL [44]. Among other activated kinases, marked induction of mTOR signaling is present in CRLF2-rearranged B-ALL as measured by increased phosphorylation of pS6, 4EBP1 and eIF4e downstream of mTORC1. Pharmacological interruption of pathway elements of PI3K/mTOR abrogated target phosphorylation [45] indicating a potential therapeutic window in this specific subgroup.

PTEN mutations are infrequent in B-ALL. PTEN is considered the counterpart to oncogenic PI3K but paradoxically it was found to be overexpressed in B-ALL blasts. However, increased PTEN levels did not lead to decreased phosphorylation of AKT but instead to increase of pAKT. This paradoxical effect was attributed to decreased PTEN phosphatase activity. CK2 is a kinase implicated in phosphorylation of PTEN thereby rendering the PTEN tumor suppressor inactive. In B-ALL, CK2 activity was increased and inhibition restored PTEN phosphatase activity with subsequent inactivation of AKT. Like PI3K inhibition, CK2 inhibition was capable of inducing apoptosis in B-ALL [41].

### T cell acute lymphoblastic leukemia (T-ALL)

Alterations of PI3K/AKT/mTOR are predominant in T-ALL with respect to other leukemia types. Frequent mutational events in T-ALL are detectable in up to 85% of all cases with homogeneous distribution over all stages of developmental arrest [46].

Functional analysis of *NOTCH1* first implicated *PTEN* in the activation of PI3K/AKT/mTOR mediating resistance to γ-secretase-inhibitors (GSI). *NOTCH1* target genes *HES1* and *MYC* were shown to negatively regulate PTEN expression and subsequent activation of PI3K/AKT was shown to induce GSI resistance [47].

Sixteen percent of T-ALL cases harbor mutations or deletions in *PTEN* leading to PTEN protein deletion [48]. Additionally, posttranslational silencing of *PTEN* is frequently observed. Like in B-ALL, PTEN phosphatase activity is diminished by high levels of CK2 and reactive oxygen species (ROS) [46]. Importantly, low levels of *PTEN* were associated with poor outcome in T-ALL, whereas its role in conjunction with *NOTCH1* mutations remains unclear [49-51].

PTEN is a major negative regulator of PI3K and loss of PTEN results in increased AKT1 kinase activity. pAKT1 has been shown to interact directly with NR3C1 preventing its translocation to the nucleus [52]. Impaired nuclear activity of NR3C1 leads to impaired glucocorticoid-induced apoptosis. Therefore, AKT1 propagates glucocorticoid resistance, which is an important indicator of therapeutic failure in T-ALL.

In PTEN-deficient solid cancers (of the brain, breast and prostate), isoform specific RNA interference identified PI3Kβ as essential for cellular growth [53], but in PTEN deficient T-ALL subtype specific *in vitro* inhibition of PI3Kβ failed to effectively inhibit downstream signaling of the PI3K/AKT/mTOR network [54]. In a mouse model of T-ALL with PTEN deficiency, both PI3Kδ and PI3Kγ supported leukemogenesis and additional silencing of these two isoforms of PI3K was capable of suppressing tumor formation [55].

Other modes of activation than *PTEN* include upstream signals feeding into PI3K/AKT/mTOR. IL-7R and IGF-1R signaling have been repeatedly implicated in playing central roles in activating PI3K/AKT/mTOR in T-ALL [56]. IGF-1R is expressed in human T-ALL and its activation by IGF-1 induces AKT activation and growth advantage. Phosphorylation of AKT is reversible upon treatment with BMS-536924, a small molecule inhibiting IGF-1R [57] and upon treatment with GSI. IGF-1R signaling is therefore sustained by NOTCH1 in T-ALL. Moreover, recent data suggest that the long non-coding RNAs (lncRNA) LUNAR1 plays a central role for the interplay of NOTCH1-IGF-1 interactions providing yet another mechanistic model for upstream activation of PI3K/AKT/mTOR signaling [58].

### Leukemia initiating cells (LIC)

There is growing evidence for a significant role of PI3K/AKT/mTOR signaling in LIC. LICs were first described in AML as a rare population enriched in CD34^+^/CD38^−^ cells with a frequency of 1:250.000 cells [59]. Later, such cells were also characterized in ALL through their capacity for engraftment and self-renewal in sequential (xeno-) transplantation. While T-cell LICs were identified within CD34^+^/CD4^−^ and CD34^+^/CD7^−^ cells respectively and B-cell LICs enriched in CD45^+^/CD19^+^ populations, the exact rate of occurrence remains controversial [60-63]. However, a common feature of LICs appears to be their resemblance to hematopoietic stem cells (HSC). For example, gene expression profiling of LICs and HSCs reveal a specific signature that independently predicted patient survival in AML [64]. It is clinically relevant to note, that both LICs and HSCs are mostly quiescent and therefore frequently insensitive to chemotherapy. Hence, relapse of acute leukemia potentially arises from LICs and the identification of distinguishing features of LICs and HSCs bears significant therapeutic potential.

Aberrant activation of PI3K/AKT/mTOR pathway has been identified as a feature of LICs in acute leukemia [65]. For instance, PTEN deletion induced leukemogenesis in a murine model. Moreover, loss of PTEN depletes absolute numbers of HSCs while increasing the frequency of LICs as PTEN^−^ leukemia was transplantable. Treatment with the mTORC1 inhibitor rapamycin achieved a reduction of leukemic burden as well as prolonging survival in diseased mice implying PI3K/AKT/mTOR signaling in leukemogenesis of PTEN^−^ acute leukemia. Importantly, rapamycin treatment abrogated the capacity to induce leukemia after secondary transplantation suggesting successful elimination of LICs. As for the depletion of HSCs, rapamycin induced an increase of HSCs with consecutive reconstitution of myeloid and lymphatic progenitors [66].

Both knock-out of raptor and rictor, essential elements of mTORC1 and mTORC2 respectively, prolonged survival in PTEN knock-out leukemic mouse models [67,68]. In fact, raptor deficiency alone in a murine model mimicking AML was unable to prevent leukemia, but suppressed leukemia progression through enhanced apoptosis in a subset of (differentiated) cells. Limited dilution assays performed with raptor-deficient undifferentiated cells demonstrated that mTORC1 was essential for leukemia initiation [69].

In a cell transplantation based zebrafish model of T-ALL, functional differences between leukemic clones were characterized by differences in growth rate, latency, leukemia propagating potential and therapy resistance. Clonal evolution led to the development of T-ALL clones displaying increased growth rate, resistance to glucocorticoids, decreased latency and, intriguingly, a significant increase in LIC frequency revealed by limiting dilution transplantation. Microarray data revealed that those clones displayed activation of AKT signaling. Accordingly, the AKT inhibitor MK2206 achieved a 25-fold reduction of LIC frequency in pAKT^+^ clones and leukemogenesis was impaired in some fish implying that LICs were efficiently cleared by pharmacological interruption of AKT signaling [70].

On the other hand, the PI3K/AKT/mTOR pathway possesses important physiological functions in HSCs. Conditional knock-out of mTOR led to bone marrow failure or defect multi-lineage hematopoiesis and engraftment of HSCs was diminished in lethally irradiated recipient mice [71]. The hope to specifically target LICs through pharmacological interruption of PI3K/AKT/mTOR without interfering with normal hematopoiesis will therefore be a considerable challenge for the future treatment of acute leukemia.

### Targeting PI3K/AKT/mTOR in acute leukemia

Preclinical evidence points to a significant role of PI3K/AKT/mTOR signaling for initiation and maintenance of acute leukemia. While the translation of preclinical models into viable clinical applications of PI3K/AKT/mTOR inhibition in acute leukemia is only at the early stages, the following sections will give an overview on existing preclinical and clinical data focusing on the most promising concepts (Table 2).

**Table 2:** **A selection of PI3K**/**mTOR**/**AKT inhibitors in clinical development for acute leukemia with a brief summary of preclinical or clinical data**

**PI3K**/**AKT**/**mTOR inhibitors in clinical development**				
**Compound**	**Target**	**Disease/** **Model**	**Result**	**Reference**
Buparlisib (BKM120)	pan PI3K	AML	1 of 11 responded lasting 80 days	Naval, LaKeisha et al., EHA Annual Meeting, 2014 [107]
	pan PI3K	T-ALL murine model	decreased leukemic activity	Lonetti, Antunes et al., Leukemia, 2014 [109]
BYL719	PI3K-α	AML murine model	decreased colony forming units in leukemia cells	Gritsman, Yuzugullu et al., J Clin Invest, 2014 [22]
BYL719 + MEKi	PI3K-α	AML	phase IB, ongoing	ClinicalTrials.gov Identifier: NCT01449058
BAY 80-6946	PI3K-δ and PI3K-α	NHL	phase II, ORR 40/67/83/50% in FL/CLL/MCL/PTCL	Dreyling, Morschhauser et al., ASH Annual Meeting 2013 [130]
ONC-01910 (rigosertib)	PI3K-α and PI3K-β	B-cell malignancies	phase I, ORR 0%, SD in 7 (50%) of 14 patients	Roschewski, Farooqui et al., Leukemia 2013 [131]
	PI3K-α and PI3K-β	MDS	phase I/II, CR in 4 (31%) of 13, SD in 8 (62%) of 13	Seetharam, Fan et al., Leuk Res, 2012 [126]
	PI3K-α and PI3K-β	MDS	phase I, ORR 6 (16%) of 37 patients	Komrokji, Raza et al., Br J Haematol, 2013 [132]
C87114	PI3Kδ	AML, initial diagnosis	inhibited proliferation *in vitro*; in combination with etoposide	Billottet, Grandage et al., Oncogene, 2006 [24]
CAL-101 (Idelalisib)	PI3Kδ	AML	*in vitro* 1 of 31 AML responded	Lanutti, Meadows et al., Blood, 2011 [98]
	PI3Kδ	B-ALL	*in vitro* 5 of 22 responded	Lanutti, Meadows et al., Blood, 2011 [98]
	PI3Kδ	B-CLL	phase III, ORR 81%	Furman, Sharman et al., NEJM, 2014 [93]
	PI3Kδ	indolent B-NHL	phase II, ORR 71 (57%) of 125	Gopal, Kahl et al., NEJM, 2014 [94]
CAL-130	PI3Kδ/γ	T-ALL murine model	prolonged survival	Subramaniam, Whye et al., Cancer Cell, 2012 [55]
IPI-145	PI3Kδ/γ	T-ALL cell line	induction of apoptosis *in vitro*	Huang, Proctor et al., ASH Annual Meeting, 2013 [99]
KP372-1	pan-PI3K/mTOR	AML	induction of apotosis *in vitro*	Zeng, Samudio et al., Cancer Res, 2006 [133]
PI-103	pan-PI3K/mTOR	T-ALL cell lines	inhibited proliferation in 15/15, induced apoptosis in 3/15	Shephard, Banerjee et al., Leukemia, 2013 [117]
	pan-PI3K/mTOR	T-ALL derived lymphoblasts	*in vitro* 7 of 7 responded	Chiarini, Fala, Cancer Res, 2009 [116]
BEZ235	pan-PI3K/mTOR	AML	phase I, ORR 0 (0%) of 11 patients, SD 1 (9%) of 11 patients	Wunderle, Badura et al., ASH annual meeting, 2013 [111]
	pan-PI3K/mTOR	ALL	phase I, ORR 3 (33%) of 9 patients	Wunderle, Badura et al., ASH annual meeting, 2013 [111]
Perifosine	AKT	CLL	phase II, ORR 1 (12,5%) of 8, SD 6 (75%) of 8 patients	Friedman, Lanasa et al., Leuk Lymphoma, 2014 [134]
Perifosine + UCN-01	AKT	AML	phase I, ORR 0 (0%) of 11 patients	Gojo, Perl et al., Invest New Drugs, 2013 [90]
GSK2141795 + MEK inhibitor	AKT1/2/3	several cancer types	*in vitro efficacy in* cell lines and murine models	Dumble, Crouthamel et al., PLOS One, 2014 [135]
GSK2141795 + Trametinib	AKT1/2/3	AML	phase II, ongoing	ClinicalTrials.gov Identifier: NCT01907815
MK-2206	AKT1	ALL	Reversal of glucocorticoid resistance in vitro and *in vivo*	Piovan, Yu et al., Cancer Cell, 2013 [52]
Triciribine (API-2)	AKT	AML	ORR 17 (53%) of 32, but no CR/PR	Sampath, Malik et al., Leuk Res, 2013 [91]
GSK690693	pan AKT	ALL	*in vitro* inhibition of proliferation and induction of apoptosis	Levy, Kahana et al., Blood, 2009 [136]
Sirolimus	mTORC1	AML	ORR monotherapy 4 (44%) of 9	Recher, Beyne-Rauzy, Blood, 2005 [31]
	mTORC2	AML	phase I, 6 (27%) of 27 combined with MEC	Perl, Kasner et al., Clin Cancer Res, 2009 [77]
Everolimus	mTORC2	B-ALL	phase I/II, ORR 7 (35%) of 20	Daver, Kantarjian et al., ASH annual meeting, 2013 [81]
Everolimus + chemotherapy	mTORC1	AML	phase Ib, 19 (68%) of 28 patients	Park, Chapuis et al., Leukemia, 2013 [33]
Temsirolimus	mTORC1	AML	phase II, ORR 11 (21%) of 53 patients	Amadori, Stasi et al., Br J Haematol, 2011 [79]

Rapamycin analogs, so called “rapalogs”, were among the first PI3K/AKT/mTOR-directed drugs in clinical use. Established in clinical practice, both everolimus and temsirolimus are directed towards mTORC1, which they allosterically inhibit. They were soon followed by a vast selection of experimental drugs with specificity against various components of the PI3K/AKT/mTOR signaling network.

Pan-PI3K inhibitors are capable of inhibiting all isoforms of class I *PI3K* (i.e. α, β, δ or γ). In contrast, isoform-specific inhibitors have specificity towards only one isoform of *PI3K*. The significant sequence homology between *PI3K* and *mTOR* has allowed the design of dual inhibitors of class I PI3K and mTOR. In contrast to the first generation of rapalogs, newer mTOR inhibitors are capable of both inhibiting mTORC1 and mTORC2. Furthermore, allosteric or catalytic inhibitors targeting AKT have been introduced in clinical trials.

This plethora of active agents makes the PI3K/AKT/mTOR perhaps the most druggable pathway in cancer medicine. At the same time, it constitutes significant challenges to implement rational schemes to identify the best match of genomic context, choice of inhibitor and combinational partner.

### mTOR inhibitors

Rapamycin was first isolated from soil samples from the Easter Islands (Rapa Nui) and later found to exhibit both cytostatic [72] and immunosuppressive properties [73]. mTOR inhibitors like temsirolimus or everolimus have since been introduced into clinical practice representing the first class of PI3K/AKT/mTOR-directed therapies. The so-called rapalogs exert their biological functions by binding of the protein folding chaperone FKBP12, which in case of rapamycin directly inhibits the function of mTORC1 [74]. This allosteric inhibition mechanism should render rapalogs ineffective in targeting other PI3K-related kinase (PIKK) family members, although some activity was observed towards mTORC2 at prolonged exposure in AML [75].

In 2005, the mTORC1 inhibitor rapamycin (also known as sirolimus) displayed antileukemic effects in relapsed AML, when four out of nine patients responded to monotherapy [76]. A later phase I study of rapamycin in relapsed/refractory AML with the chemotherapy regimen MEC (i.e. mitoxantrone, etoposide, cytarabine) produced an overall response rate of 22% (i.e. 6 of 27 subjects) [77]. In principle, evaluation of response of phase I clinical trials was problematic, as the objective of phase I trials was dose finding. However, temsirolimus, another mTORC1 inhibitor approved for the treatment of mantle cell lymphoma in Europe [78], failed to induce higher remission rates in a phase II study, whereas in elderly relapsed or refractory AML patients 11 (21%) of 53 evaluable patients in combination with clofarabine responded. Taken together, these results failed to provide a significant improvement of response. Nevertheless, pharmacodynamics revealed that effective downstream inhibition of S6 ribosomal protein (S6RP) phosphorylation correlated with response. The response rate of patients with >50% inhibition of pS6RP was 75% compared to 0% in patients with <50% pS6RP inhibition suggesting clinical activity of temsirolimus in AML [79].

In a more recent GOELAMS phase Ib study, the mTORC1 inhibitor everolimus was tested in first relapse in a younger cohort of AML patients (i.e. <65 years) in combination with intensive chemotherapy. While 19 (68%) of 28 patients achieved CR, stronger downstream inhibition (of p70S6K) was again associated to higher CR rates [33].

This illustrates an important caveat of targeted therapies; in addition to effective inhibition of molecular targets, sufficient downstream inhibition of effector molecules is necessary to produce clinical benefits. Inhibition of S6RP phosphorylation, among others, turned out to be a predictive marker of efficient pathway inhibition and its measurement is feasible in the clinical routine [80].

In B-ALL, the combination of intensive chemotherapy (Hyper-CVAD) plus the mTORC1 inhibitor everolimus in relapsed/refractory ALL exhibited acceptable toxicities. Downstream effector inhibition was observed in seven of 10 patients analyzed for p-pS6K. Six (30%) of 20 patients enrolled to this phase I/II trial achieved complete remission [81] compared to a CR rate of 18% - 33% reported elsewhere for patients in second relapse [7,82]. Although this did not constitute a major improvement of response, pharmacokinetics revealed that CR patients had significantly higher AUC of everolimus than those with PR/PD suggesting anti-leukemic activity of everolimus in B-ALL.

Clinical experience of mTOR inhibition in T-ALL is limited, although recent data implicates mTOR in the development of early T-cell progenitors (ETP) and T-ALL [83].

Resistance to glucocorticoids in ALL is associated to adverse outcome. As targeted therapy might be capable of overcoming resistance to standard treatment, the mTORC1 inhibitor rapamycin in combination with dexamethasone induced cell cycle arrest and apoptosis in a wide range of ALL cell lines. PTEN^null^ cell lines were more sensitive to rapamycin and dexamethasone. In a murine PTEN mutated xenograft, the combination prolonged event-free survival of mice. The important role of mTOR and its inhibition was further reinforced, when deficiency of Raptor, an essential part of mTORC1, induced eradication of leukemia in a murine model of T-ALL. In contrast, pharmacological inhibition of mTORC1 by rapamycin prolonged survival in T-ALL bearing mice, but rapamycin-insensitive clones would eventually prevail resulting in failure of disease eradication [83].

**Figure 1:**
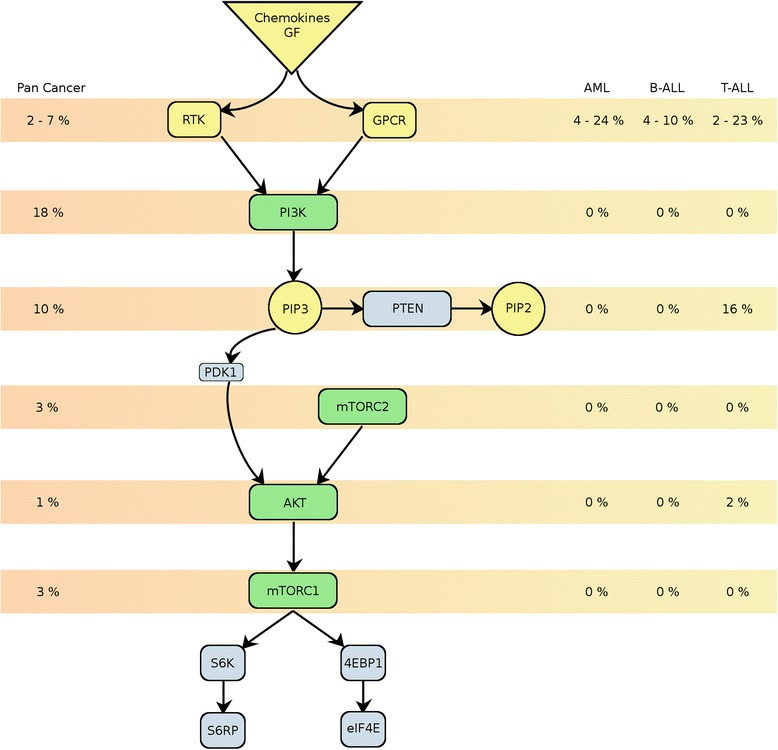
**Schematic diagram of a simplified PI3K/** **AKT/** **mTOR network comparing the mutational landscape of PI3K/** **AKT/** **mTOR components in acute leukemia versus pan**-**cancer.** Mutational frequencies were obtained from COSMIC v69 repository [48].

Bearing the problem of rapamycin resistance in mind, mTOR inhibitors with specificity to both mTOR complexes, so called “TORKinhibs”, have been introduced. A common feature of these second generation mTOR inhibitors is their capacity to decrease AKT activity through inhibition of mTORC2 (Figure 1). As catalytic inhibitors of the mTOR kinase, they exert differential antiproliferative and apoptotic properties compared to rapalog derivates [84]. Unlike rapalogs, TORKinhibs abolish all mTORC1 functions, including the control of 4EBP1 phosphorylation and protein translation. As an example, the TORKinhib MLN0128 (formerly known as INK128) suppressed proliferation of B-ALL cell lines [85] and TORKinhibs like Ku-0063794 [86], AZD2014 [87] or CC-223 [88] have demonstrated in vitro cytotoxicity in a range of cancers. Therefore, TORKinhibs are important candidates for future implementation in treatment protocols of acute leukemia. However, at the time of this review, no clinical data on *in vivo* efficacy has been reported.

### AKT inhibitors

AKT is seen by many as the central player of the PI3K/AKT/mTOR circuit, but until now AKT inhibitors have been rarely tested in either preclinical nor clinical settings.

UCN-01 was shown to induce S-phase arrest and apoptosis in combination with cytarabine in the AML cell line ML-1. In primary AML cells, the addition of UCN-01 to cytarabine was cytotoxic, while in healthy hematopoietic progenitors no toxicity was observed. These observations were accompanied by a strong decrease in AKT phosphorylation implicating UCN-01 as an AKT inhibitor [89]. Identified later as a potent inhibitor of PDK1, UCN-01 was used in combination with perifosine, a direct inhibitor of AKT. In a phase I trial, thirteen patients with advanced acute leukemia were treated at different dose levels. Overall, toxicity was alarmingly high (pericardial effusion, hyperglycemia, pneumonitis etc.) and while downstream RPS6 phosphorylation was decreased, no significant AKT inhibition was observed. Clinical activity was disappointing and no objective response recorded [90]. The problem of toxicity was overcome in a phase I clinical trial with triciribine phosphate monohydrate (TCN-PM) in advanced AML. AKT inhibition was determined in eight patients. TCN-PM decreased p-AKT in all three patients with increased baseline levels of p-AKT, while no change was observed in the others. Out of 43 patients enrolled to the trial 32 were evaluable for response. No complete or partial response was registered, although 17 patients had stable disease and three AML patients displayed a bone marrow blast reduction of 50% or higher [91].

### Isotype specific PI3K inhibition

While PI3Kα and PI3Kβ subunits are expressed in nearly all cells of the human body, the PI3Kδ and PI3Kγ subunits are found almost exclusively on leucocytes [16] and therefore they constitute an attractive target for hematopoietic malignancies [92]. Indeed, idelalisib, an inhibitor of PI3Kδ, is exceptionally effective as monotherapy in chronic lymphocytic leukemia (CLL) and indolent Non-Hodgkin-Lymphoma (NHL) [93,94]. However, the presumed mode of action, i.e. suppression of pro-survival signals via inhibition of the δ subunit of PI3K, does not reflect its clinical activity with rapid decrease of lymph nodes and lymphocytosis. Instead, additional tumor microenvironment-dependent factors may be responsible for its pattern of clinical activity. For instance, idelalisib down-regulates secretion of chemokines in stromal co-cultures of CLL cells and inhibits BCR-dependent ERK activation [95].

Although these mechanisms might be present only in mature B-cell entities such as NHL and CLL, idelalisib increased apoptosis in B-ALL cell lines without depleting the reservoir of normal T cells [96]. Growth inhibition of idelalisib in B-ALL cell lines was associated to the presence of pre-BCR and gene expression profiling revealed down-regulation of MAP kinase signaling upon treatment with idelalisib in compliance with data from Hoellenriegel et al. in CLL [97]. Primary B-ALL cells from patients exposed to idelalisib exhibited sensitivity in 5 (23%) of 22 patients [98]. Taken together, idelalisib has grand anti-leukemic potential in B-ALL, albeit data on clinical efficacy are outstanding at the time of this review.

PI3Kδ and PI3Kγare also instrumental for the maturation of T-cells. In a PTEN-deficient murine model, PI3Kγ and PI3Kδ alone are capable of promoting T cell leukemia. The PI3Kδ/γ-specific inhibitor CAL-130 confirmed the addiction of PTEN^null^ T-ALL on PI3Kδ and PI3Kγ by reducing tumor burden and prolonging survival in PTEN^null^ T-ALL mice. In primary human T-ALL cells, combined inhibition of PI3Kδ/γ reduced tumor viability in PTEN^null^ samples. Moreover, in PTEN^+^ samples, presence of pAKT predicted tumor response upon CAL-130 treatment [55]. These observations were further supported by a study comparing PI3K β-, δ-, γ- and δ/γ - isoform inhibiting compounds. In a PTEN^null^ setting of the human T-ALL cell line Loucy, the PI3Kδ/γ inhibitor IPI-145 yielded the most robust growth inhibition and induction of apoptosis [99]. A phase I trial of IPI-145 is ongoing including T-ALL patients and with recruitment already complete, data analysis is ongoing at the time of this review and will be completed by the end of this year.

Like in ALL, PI3Kδ is likely an attractive target in AML. PI3Kδ is homogeneously expressed at high levels in AML blasts and IC87114, a PI3Kδ inhibitor, demonstrated robust AKT inhibition. IC87114 decreased proliferation of AML blasts while sparing normal hematopoietic progenitors [23]. In primary AML cells, IC87114 exhibited in vitro activity in combination with etoposide that was greater than each of the two drugs alone [24]. On the other hand, Idelalisib, another inhibitor of PI3Kδ, with remarkable activity in B cell lymphoma [93,94] had limited *in vitro* activity in AML in only one of 31 evaluated patients [98]. However, a systematic clinical assessment of PI3Kδ inhibition in AML is unavailable at the time of this review.

Multiple lines of evidence exist for how PI3K/AKT/mTOR inhibition alone induces shunting of survival and proliferation signals [100-102]. As a consequence, many current clinical trials focus on combination therapy.

In a KRAS hyperactive murine model of AML, knockout of PI3K subunit α prolonged survival. Pharmacological interruption with BYL719, a selective PI3Kα inhibitor, impaired bone marrow colony formation of KRAS mutated leukemic cells. Moreover, synergistic effects were reported for the additional use of MEK inhibitor MEK162 capable of reducing proliferation and abrogating leukemia in this AML xenograft [22].

Consequentially, one recruiting trial for subtype-specific PI3K inhibition in acute leukemia interrogates the efficacy of the PI3Kα inhibitor BYL719 in combination with MEK162 (clinicaltrials.gov, ID#NCT01449058). As results of phase I trials of the PI3Kδ inhibitor TGR-1202 for hematological cancers are pending (clinicaltrials.gov, ID# NCT02268851, NCT01767766), efficacy data of PI3Kδ inhibition in acute leukemia will be particularly interesting given the compelling preclinical rationale for its implementation [23,24].

Subtype specific inhibition may exert clinical efficacy in acute leukemia while exhibiting a more favorable toxicity profile when compared to agents targeting multiple isoforms. PI3Kα is the primary mediator of insulin action via the IGF-1 and in vivo studies on mice demonstrated significant interference with glucose metabolism as well as skeletal alterations [103]. accordingly, the clinical use of PI3Kα inhibitors has produced high rates of hyperglycemia [104]. As the PI3Kδ subunit is restricted to leukocytes, immunomodulatory side effects were predicted upon PI3Kδ inhibition. Indeed, ≥ grade III pneumonia or Neutropenia occurred in 20 and 11% in CLL [105] and slightly less frequent in mantle cell lymphoma (10% and 10%) [106]. experimental evidence suggested that PI3Kδ inhibition with IC87114 may spare normal hematopoiesis [23,24].

With PI3Kδ being the most likely isoform of PI3K to target in acute leukemia, myelosuppression at efficacious doses was manageable demonstrating that a therapeutic window for PI3Kδ isotype specific inhibition may exist.

### Pan-PI3K inhibition

Isoform specific inhibition of PI3K promises more specificity with a more favorable toxicity profile, but produces a “bottleneck” addiction of the targeted pathway. Therefore, targeting all isoforms of PI3K with pan-PI3K inhibitors may have a broader anti-leukemic efficacy at the expense of a less favorable toxicity profile.

Buparlisib (BKM120) is likely the pan-PI3K inhibitor with the most advanced clinical development. In a phase I/II trial in relapsed/refractory AML, buparlisib displayed an acceptable toxicity profile with mostly mild dysphagia, mucositis, and elevated serum bilirubin, but, notably, no elevated serum glucose. The maximum tolerated dose (MTD) exhibited sufficient target effector down-regulation, which was measured as inhibition of pS6RP and FOXO3 phosphorylation in approximately two thirds of the patients. Clinical efficacy, however, was modest with only one of 11 patients who achieved stable disease with a duration of 80 days. A decrease of both p-pS6RP (by 45%) and FOXO3 (100%) was reported [107]. Thus, it’s evident that target effector down-regulation may be a necessary requirement for clinical efficacy, but fails to predict meaningful clinical responses.

A large analysis of T-ALL cell lines with and without loss of PTEN, for instance, failed to detect superiority of subtype specific versus pan-PI3K inhibition. In fact, the two pan-PI3K inhibitors BKM120 and ZSTK454 exerted meaningful effects on cell viability and apoptosis, whereas p110α-, p110β-, p110δ, p110γ- and p110δ/γ-selective inhibitors did not [108]. Moreover, the pan-PI3K inhibitor buparlisib (BKM120) displayed antileukemic effects in primary patient T-lymphoblasts and prolonged survival in subcutaneously injected murine models of T-ALL [109].

A major drawback of targeted therapy is secondary drug resistance. Although primary leukemia cells display sensitivity towards the pan-PI3K inhibitor GFC-0941, all mice eventually relapsed with resistant tumor outgrowths. PD analysis revealed paradoxical activation of PI3K signaling and interestingly, resistant clones exhibited decreased *NOTCH1* activity and resistance to GSI [110].

### Dual PI3K/mTOR inhibition

Drug resistance is frequently observed upon STI monotherapy and therefore additional principles are needed to reinforce therapeutic success. Combination therapy does not only consist of combining targeted therapy with chemotherapy, but also of simultaneous application of various types of targeted therapy. Sequence homology of diverse kinases has allowed developing molecules with specificity towards both PI3K and mTOR while affinity to the rest of the human kinome is limited.

Clinical activity of “vertical inhibition” of PI3K/AKT/mTOR was seen with BEZ235, a dual pan-PI3K and mTOR inhibitor, efficiently inhibiting 4EBP1 and protein translation in AML blasts reducing cell growth and inducing apoptosis [30]. The same BEZ235 was tested in a phase I study of 22 patients, including 11 patients with relapsed/refractory AML. Only one patient achieved stable disease of four month duration. Although the primary goal of this trial was dose finding, the response data so far suggests little activity of BEZ235 in AML [111].

Ph-like B-ALL is a subtype of B-ALL with a kinase activated gene expression profile resembling Ph^+^ B-ALL, yet without BCR-ABL fusion [44]. A range of diverse PI3K/mTOR/AKT inhibitors were tested in patient-derived Ph-like B-ALL cells transplanted into NOD-SCID-γ-null (NSG) mice. The drugs tested included inhibitors of PI3Kα, PI3Kδ, both PI3K and mTOR and mTORC1/mTORC2. Pharmacodynamics revealed inhibition of pS6 and p4EBP1 by the dual PI3K/mTOR inhibitor PKI587, but little effect on upstream elements such as AKT. Nevertheless, PKI587 induced the biggest decrease of leukemic activity in blood, bone marrow and spleen in this murine model of B-ALL [112].

In a similar setup, a range of PI3K/AKT/mTOR-directed therapies (e.g. pan-PI3K inhibitors, mTORC1 inhibitors, mTORC1/2 inhibitors and dual PI3K/mTORC1/2 inhibitors) have been tested in long-term cultures of patient-derived B-ALL cells. Here, too, combined inhibition of PI3K and mTORC1/2 exhibited the greatest antileukemic activity [113].

Additional evidence for efficacy from dual PI3K/mTOR inhibition in B-ALL stems from experiments in Ph^+^-B-ALL with BEZ235, a dual pan-PI3K/mTORC1/mTORC2 inhibitor. Upon treatment of Ph^+^ cell lines, inhibition of p-4E-BP1, p70S6K and, notably, pAKT was registered. Furthermore, synergistic effects in inhibiting tumor growth of BEZ235 with nilotinib were reported in a xenograft model of bcr-abl mutant cells [114]. In Ph^−^-B-ALL, too, BEZ235 displayed *in vitro* activity in combination with chemotherapy such as doxorubicin, cytarabine or dexamethasone [115].

However, clinical activity of BEZ235 in a German phase I trial with 22 patients was rather disappointing. In addition to dose finding, the reported overall response rate in B-ALL was 33%, i.e. three of nine patients. The complete remission was observed in a patient with pro-B-ALL after allogeneic stem cell transplantation and two partial remissions with hematological improvement in both Ph^+^- and Ph^−^- B-ALL [111]. Therefore, dual PI3K/mTOR inhibition seems to be efficient only in a fraction of B-ALL patients. Future efforts must be directed towards identifying these patients in order to bring to full use dual PI3K/mTOR inhibition in B-ALL.

PI-103 is a synthetic molecule with sensitivity to both PI3K and mTOR kinases. In T-ALL cell lines, PI-103 produced synergistic effects in impairing proliferation in combination with chemotherapy. In patient derived T cell lymphoblasts, PI-103 induced cytotoxicity in all seven patient samples [116]. PI-103 treated cells revealed up-regulation of NOTCH1 target genes, including c-MYC by microarray analysis. The combination of PI-103 with either GSI or a c-MYC inhibitor enhanced cell cycle arrest and achieved cell death [117].

### Drawbacks and future directions

Given the overall convincing preclinical data on PI3K/AKT/mTOR signaling in acute leukemia, the results of clinical trials have been rather disappointing. Currently, much effort is being made to identify drawbacks of clinical trials of the past to improve efficacy of pharmacological interruption of PI3K/AKT/mTOR in the future.

For instance, efforts to understand the lack of meaningful antileukemic activity of rapalogs have revealed two major mechanistic explanations: inhibition of mTORC1 by rapalogs inhibits p70S6K transmitting a negative IRS1-dependent feedback leading to up-regulation of RTKs and PI3K/AKT activity [17,118]. The second major mechanism of resistance to mTORC1 inhibition is the failure to abrogate all mTORC1 functions leaving mTORC1-mediated phosphorylation of 4EBP1 (at S65) and consecutive translational deregulation unimpaired [72].

The dual PI3K/mTOR inhibitor NVP-BEZ235 was capable to overcome these mechanisms and displayed potent antitumor activity in breast cancer *in vitro*. However, as pointed out above, clinical efficacy of dual PI3K/mTOR inhibition in acute leukemia was limited.

The lack of convincing clinical activity of mTOR-directed therapies might be due to yet another phenomenon that was recently reviewed by Medvetz and colleagues [119]. Hyperactive mTORC1 activity might be a mechanism to counterbalance bioenergetic instability as a result of upstream lesions rather than a cancer promoting process per se. Instead of abolishing the mTORC1 signal, exploitation of the resulting metabolic vulnerabilities, such as glucose, glutamine or autophagy addiction may be a worthwhile strategy.

Nevertheless, elucidating the details of PI3K/AKT/mTOR signalling has provided important insights that will help to increase efficacy of STI. In primary AML samples, Bertacchini and colleagues found that selective inhibition of mTOR and AKT led to paradoxical phosphorylation of AKT in 70% of all cases resulting in stabilization of PI3K/AKT/mTOR downstream effectors, such as IRS-1 and FOXO. RTKs were also up-regulated, thereby bypassing the effect of PI3K/AKT/mTOR inhibition [120]. Therefore, future clinical trials must investigate the efficacy of combining TKI and PI3K/AKT/mTOR inhibition *in vivo*, as the combination has displayed significant synergistic effects in preclinical experiments.

However, the combination of PI3K/AKT/mTOR directed STI with TKI is not the only rationale. The clinicaltrials.gov database currently enlists 76 recruiting clinical trials assessing the role of combination therapy of PI3K inhibition in advanced cancers. Nearly all those trials include solid, non-hematological cancers and assessment in acute leukemia is needed.

Rational strategies for combination therapy in acute leukemia are beginning to surface.

Vachhani and coworkers, for example, proposed the rational combination of PI3K/AKT/mTOR directed STI in combination with BCL-2/-xL antagonists [101]. PI3K/AKT/mTOR signaling is closely interconnected with BCL-2 family proteins. In fact, AKT up-regulates BCL-2 and MCL-1 via CREB (cyclic adenosine monophosphate response element binding protein) [121,122] and theoretically combined disruption of PI3K/AKT/mTOR and anti-apoptotic functions promises substantial synergistic effects. Indeed, dual knockdown of BCL-2 and BCL-xL increased sensitivity of PI3K/AKT/mTOR inhibition in human AML cell lines and knockdown of AKT, on this end, increased lethality of the BCL-2/-xL inhibitor ABT-737. In primary AML samples, co-administration of BEZ235 and ABT-737 exerted increased lethality in all four samples analyzed with basal AKT activation and the combination increased survival in a murine subcutaneous xenograft model [123]. As both types of targeted therapies lack single agent efficacy, perhaps their combination will achieve clinically significant antileukemic effects.

The combination of PI3K/AKT/mTOR and MAPK inhibition is another important thread to be followed. Mutant proto-oncogenes are frequently localized at the cell membrane feeding growth signals into “downstream” signaling pathways, such as PI3K/AKT/mTOR and MAPK. Combined inactivation of both of these signaling networks can sufficiently interrupt oncogenic signals in lung cancer [124]. In that regard, clinical testing of BYL710 co-administered with MEK162 is currently under way in AML patients (clinicaltrials.gov, ID#NCT01449058).

Lastly, the combined inhibitor of PI3K and the polo-like kinase 1 rigosertib may constitute a complimentary approach to PI3K/AKT/mTOR inhibition [125]. Rather than competing for the ATP binding socket, this allosteric inhibitor of substrate binding has demonstrated proliferative arrest and induction of apoptosis in myeloblasts and clinical trials in myelodysplastic syndrom are encouraging [126], where a benefit in overall survival was recently reported in patients with primary resistance to hypomethylating agents and those with IPSS very high risk [127].

The outstanding success of TKI in BCR-ABL^+^ CML has established the idea that STI is most efficient when a “master node” within a signaling network is targeted. As for the PI3K/AKT/mTOR network, perhaps PI3Kδ/γ constitutes such a target. In Non-Hodgkin-Lymphoma (NHL) the PI3K subtype specific inhibitor CAL-101 has achieved remarkable therapeutic efficacy [93,94,96] and mechanistic explanation for its anti-tumor activity are promising. For instance, PI3Kδ^D910A^ mutated mice displayed a reduced tumor incidence from 97% in wildtype to 65% in mutated cases, when inoculated with diverse cancer cell lines resembling melanoma, lung cancer, thymoma or breast cancer. With PI3Kδ deletion selective for regulatory T cells (T_reg_), only 40% of mice developed tumors indicating the significance of PI3Kδ for T_reg_ mediated immune tolerance. While inhibitory effects of PI3Kδ deletion on cytotoxic T cells (CTL) were also observed, the overriding effect on T_reg_ exhibited enough anti-tumor immunity, as some PI3Kδ^D910A^ mice initially developed cancer, but displayed regression after two weeks indicating remaining anti-tumor activity of CTLs [128]. Along with compelling evidence, that PI3Kδ may constitute the most relevant target in acute leukemia, these data provide a rationale for enhancing the indication of PI3Kδ selective inhibitors alone or possibly in combination with other strategies to promote tumor-specific immune responses (e.g. vaccines, adoptive cell therapy, PD-1/PD-L1 blockade, CTLA4 blockade).

However, there are signs that some combinations will prove to be counterproductive. Combining lymphocyte stimulating therapies like PD-1/PD-L1 blockade with PI3K inhibition might induce suppression of the very T cell function that anti-PD-1/PD-L1 treatment restored in the first place [129].

More work in deciphering the molecular circuits at work in cancerogenesis is necessary to bring to full use the possibilities of targeted therapy.

## Conclusions

Although there is an encouraging signal for PI3K/AKT/mTOR activation in acute leukemia, clinical efficacy of inhibition has been disappointingly modest so far and it is becoming clear that as monotherapy, pharmacological interruption of PI3K/AKT/mTOR will only be successful in a subgroup of patients. We predict that PI3Kδ would be the most efficient target in acute leukemia and additionally emphasize that vertical inhibition of various components of PI3K/AKT/mTOR (e.g. PI3K and mTOR) will further enhance efficacy of STI. In order to identify patients that would benefit most, it will be pivotal to further unravel the PI3K/AKT/mTOR-mediated molecular signatures of cellular growth, survival and metabolism including the effects of its inhibition. This process has already produced meaningful arguments for disease stratification and combinational therapies and will surely continue to do so in the future.

However, molecular stratification will produce a decreasing cohort size ultimately challenging traditional pillars of evidence based medicine like randomized clinical trials. Clinical testing will require newer design such as “pick the winner” or umbrella trials that promise faster, but yet scientifically sound implementation of what is already a large repertoire of genetically driven, targeted therapy.

We predict that PI3K/AKT/mTOR inhibition will constitute a valuable tool in an ever growing arsenal of drugs with anti-leukemic activity, albeit likely only in a subset of patients with acute leukemia.

## References

[1] Kandoth C, McLellan MD, Vandin F, Ye K, Niu B, Lu C (2013). Mutational landscape and significance across 12 major cancer types. Nature.

[2] Hudes G, Carducci M, Tomczak P, Dutcher J, Figlin R, Kapoor A (2007). Temsirolimus, interferon alfa, or both for advanced renal-cell carcinoma. N Engl J Med.

[3] Röllig CM, CRöllig C, Müller-Tidow C, Hüttmann A, Noppeney R, Kunzmann V (2014). Sorafenib versus placebo in addition to standard therapy in younger patients with newly diagnosed acute myeloid leukemia: results from 267 patients treated in the randomized placebo-controlled SAL-soraml trial. ASH annual meeting and exposition 2014.

[4] Elert E (2013). Living with leukaemia. Nature.

[5] Wattad M (2014). Impact of the composition of salvage regimens on response and overall survival in primary refractory acute myeloid leukemia. EHA 19th, June 12–15, 2014. vol. ABSSUB-5216.

[6] Gokbuget N, Hoelzer D (2009). Treatment of adult acute lymphoblastic leukemia. Semin Hematol.

[7] Gokbuget N, Stanze D, Beck J, Diedrich H, Horst HA, Huttmann A (2012). Outcome of relapsed adult lymphoblastic leukemia depends on response to salvage chemotherapy, prognostic factors, and performance of stem cell transplantation. Blood.

[8] Freeman SD, Virgo P, Couzens S, Grimwade D, Russell N, Hills RK (2013). Prognostic relevance of treatment response measured by flow cytometric residual disease detection in older patients with acute myeloid leukemia. J Clin Oncol.

[9] Rowley JD (1973). Letter: a new consistent chromosomal abnormality in chronic myelogenous leukaemia identified by quinacrine fluorescence and Giemsa staining. Nature.

[10] Lee HJ, Thompson JE, Wang ES, Wetzler M (2011). Philadelphia chromosome-positive acute lymphoblastic leukemia: current treatment and future perspectives. Cancer.

[11] Benjamini O, Dumlao TL, Kantarjian H, O'Brien S, Garcia-Manero G, Faderl S (2014). Phase II trial of hyper CVAD and dasatinib in patients with relapsed Philadelphia chromosome positive acute lymphoblastic leukemia or blast phase chronic myeloid leukemia. Am J Hematol.

[12] Chomienne C, Cornic M, Castaigne S, Lefebvre P, de The H, Dejean A (1991). Biological parameters of the efficiency of retinoic acid in acute leukemia. C R Seances Soc Biol Fil.

[13] Lo-Coco F, Avvisati G, Vignetti M, Thiede C, Orlando SM, Iacobelli S (2013). Retinoic acid and arsenic trioxide for acute promyelocytic leukemia. N Engl J Med.

[14] Buitenhuis M, Coffer PJ (2009). The role of the PI3K-PKB signaling module in regulation of hematopoiesis. Cell Cycle.

[15] Fruman DA, Rommel C (2014). PI3K and cancer: lessons, challenges and opportunities. Nat Rev Drug Discov.

[16] Vanhaesebroeck B, Welham MJ, Kotani K, Stein R, Warne PH, Zvelebil MJ (1997). P110delta, a novel phosphoinositide 3-kinase in leukocytes. Proc Natl Acad Sci U S A.

[17] Tamburini J, Chapuis N, Bardet V, Park S, Sujobert P, Willems L (2008). Mammalian target of rapamycin (mTOR) inhibition activates phosphatidylinositol 3-kinase/Akt by up-regulating insulin-like growth factor-1 receptor signaling in acute myeloid leukemia: rationale for therapeutic inhibition of both pathways. Blood.

[18] Vanhaesebroeck B, Stephens L, Hawkins P (2012). PI3K signalling: the path to discovery and understanding. Nat Rev Mol Cell Biol.

[19] Jimenez C, Jones DR, Rodriguez-Viciana P, Gonzalez-Garcia A, Leonardo E, Wennstrom S (1998). Identification and characterization of a new oncogene derived from the regulatory subunit of phosphoinositide 3-kinase. EMBO J.

[20] Kubota Y, Ohnishi H, Kitanaka A, Ishida T, Tanaka T (2004). Constitutive activation of PI3K is involved in the spontaneous proliferation of primary acute myeloid leukemia cells: direct evidence of PI3K activation. Leukemia.

[21] Xu Q, Simpson SE, Scialla TJ, Bagg A, Carroll M (2003). Survival of acute myeloid leukemia cells requires PI3 kinase activation. Blood.

[22] Gritsman K, Yuzugullu H, Von T, Yan H, Clayton L, Fritsch C (2014). Hematopoiesis and RAS-driven myeloid leukemia differentially require PI3K isoform p110alpha. J Clin Invest.

[23] Sujobert P, Bardet V, Cornillet-Lefebvre P, Hayflick JS, Prie N, Verdier F (2005). Essential role for the p110delta isoform in phosphoinositide 3-kinase activation and cell proliferation in acute myeloid leukemia. Blood.

[24] Billottet C, Grandage VL, Gale RE, Quattropani A, Rommel C, Vanhaesebroeck B (2006). A selective inhibitor of the p110delta isoform of PI 3-kinase inhibits AML cell proliferation and survival and increases the cytotoxic effects of VP16. Oncogene.

[25] Tamburini J, Elie C, Bardet V, Chapuis N, Park S, Broet P (2007). Constitutive phosphoinositide 3-kinase/Akt activation represents a favorable prognostic factor in de novo acute myelogenous leukemia patients. Blood.

[26] Min YH, Eom JI, Cheong JW, Maeng HO, Kim JY, Jeung HK (2003). Constitutive phosphorylation of Akt/PKB protein in acute myeloid leukemia: its significance as a prognostic variable. Leukemia.

[27] Gallay N, Dos Santos C, Cuzin L, Bousquet M, Simmonet Gouy V, Chaussade C (2009). The level of AKT phosphorylation on threonine 308 but not on serine 473 is associated with high-risk cytogenetics and predicts poor overall survival in acute myeloid leukaemia. Leukemia.

[28] Kornblau SM, Womble M, Qiu YH, Jackson CE, Chen W, Konopleva M (2006). Simultaneous activation of multiple signal transduction pathways confers poor prognosis in acute myelogenous leukemia. Blood.

[29] Brandts CH, Sargin B, Rode M, Biermann C, Lindtner B, Schwable J (2005). Constitutive activation of Akt by Flt3 internal tandem duplications is necessary for increased survival, proliferation, and myeloid transformation. Cancer Res.

[30] Chapuis N, Tamburini J, Cornillet-Lefebvre P, Gillot L, Bardet V, Willems L (2010). Autocrine IGF-1/IGF-1R signaling is responsible for constitutive PI3K/Akt activation in acute myeloid leukemia: therapeutic value of neutralizing anti-IGF-1R antibody. Haematologica.

[31] Recher C, Dos Santos C, Demur C, Payrastre B (2005). mTOR, a new therapeutic target in acute myeloid leukemia. Cell Cycle.

[32] Chow S, Minden MD, Hedley DW (2006). Constitutive phosphorylation of the S6 ribosomal protein via mTOR and ERK signaling in the peripheral blasts of acute leukemia patients. Exp Hematol.

[33] Park S, Chapuis N, Saint Marcoux F, Recher C, Prebet T, Chevallier P (2013). A phase Ib GOELAMS study of the mTOR inhibitor RAD001 in association with chemotherapy for AML patients in first relapse. Leukemia.

[34] Ley T (2013). Genomic and epigenomic landscapes of adult de novo acute myeloid leukemia. N Engl J Med.

[35] Liu TC, Lin PM, Chang JG, Lee JP, Chen TP, Lin SF (2000). Mutation analysis of PTEN/MMAC1 in acute myeloid leukemia. Am J Hematol.

[36] Cheong JW, Eom JI, Maeng HY, Lee ST, Hahn JS, Ko YW (2003). Phosphatase and tensin homologue phosphorylation in the C-terminal regulatory domain is frequently observed in acute myeloid leukaemia and associated with poor clinical outcome. Br J Haematol.

[37] Rodriguez-Viciana P, Warne PH, Dhand R, Vanhaesebroeck B, Gout I, Fry MJ (1994). Phosphatidylinositol-3-OH kinase as a direct target of Ras. Nature.

[38] Harrison-Findik D, Susa M, Varticovski L (1995). Association of phosphatidylinositol 3-kinase with SHC in chronic myelogeneous leukemia cells. Oncogene.

[39] Kharas MG, Janes MR, Scarfone VM, Lilly MB, Knight ZA, Shokat KM (2008). Ablation of PI3K blocks BCR-ABL leukemogenesis in mice, and a dual PI3K/mTOR inhibitor prevents expansion of human BCR-ABL+ leukemia cells. J Clin Invest.

[40] Jou ST, Carpino N, Takahashi Y, Piekorz R, Chao JR, Carpino N (2002). Essential, nonredundant role for the phosphoinositide 3-kinase p110delta in signaling by the B-cell receptor complex. Mol Cell Biol.

[41] Gomes AM, Soares MV, Ribeiro P, Caldas J, Povoa V, Martins LR (2014). Adult B-cell acute lymphoblastic leukemia cells display decreased PTEN activity and constitutive hyperactivation of PI3K/Akt pathway despite high PTEN protein levels. Haematologica.

[42] Morishita N, Tsukahara H, Chayama K, Ishida T, Washio K, Miyamura T (2012). Activation of Akt is associated with poor prognosis and chemotherapeutic resistance in pediatric B-precursor acute lymphoblastic leukemia. Pediatr Blood Cancer.

[43] Brown VI, Fang J, Alcorn K, Barr R, Kim JM, Wasserman R (2003). Rapamycin is active against B-precursor leukemia in vitro and in vivo, an effect that is modulated by IL-7-mediated signaling. Proc Natl Acad Sci U S A.

[44] Roberts KG, Morin RD, Zhang J, Hirst M, Zhao Y, Su X (2012). Genetic alterations activating kinase and cytokine receptor signaling in high-risk acute lymphoblastic leukemia. Cancer Cell.

[45] Tasian SK, Doral MY, Borowitz MJ, Wood BL, Chen IM, Harvey RC (2012). Aberrant STAT5 and PI3K/mTOR pathway signaling occurs in human CRLF2-rearranged B-precursor acute lymphoblastic leukemia. Blood.

[46] Silva A, Yunes JA, Cardoso BA, Martins LR, Jotta PY, Abecasis M (2008). PTEN posttranslational inactivation and hyperactivation of the PI3K/Akt pathway sustain primary T cell leukemia viability. J Clin Invest.

[47] Palomero T, Sulis ML, Cortina M, Real PJ, Barnes K, Ciofani M (2007). Mutational loss of PTEN induces resistance to NOTCH1 inhibition in T-cell leukemia. Nat Med.

[48] COSMIC. Sharing Data from Large-scale Biological Research Projects: A System of Tripartite Responsibility. In: COSMIC repository: 2003; Fort Lauderdale, Florida, USA; 2003.

[49] Gutierrez A, Sanda T, Grebliunaite R, Carracedo A, Salmena L, Ahn Y (2009). High frequency of PTEN, PI3K, and AKT abnormalities in T-cell acute lymphoblastic leukemia. Blood.

[50] Trinquand A, Tanguy-Schmidt A, Ben Abdelali R, Lambert J, Beldjord K, Lengline E (2013). Toward a NOTCH1/FBXW7/RAS/PTEN-based oncogenetic risk classification of adult T-cell acute lymphoblastic leukemia: a Group for Research in Adult Acute Lymphoblastic Leukemia study. J Clin Oncol.

[51] Bandapalli OR, Zimmermann M, Kox C, Stanulla M, Schrappe M, Ludwig WD (2013). NOTCH1 activation clinically antagonizes the unfavorable effect of PTEN inactivation in BFM-treated children with precursor T-cell acute lymphoblastic leukemia. Haematologica.

[52] Piovan E, Yu J, Tosello V, Herranz D, Ambesi-Impiombato A, Da Silva AC (2013). Direct reversal of glucocorticoid resistance by AKT inhibition in acute lymphoblastic leukemia. Cancer Cell.

[53] Wee S, Wiederschain D, Maira SM, Loo A, Miller C, deBeaumont R (2008). PTEN-deficient cancers depend on PIK3CB. Proc Natl Acad Sci U S A.

[54] Stengel C, Jenner E, Meja K, Mayekar S, Khwaja A (2013). Proliferation of PTEN-deficient haematopoietic tumour cells is not affected by isoform-selective inhibition of p110 PI3-kinase and requires blockade of all class 1 PI3K activity. Br J Haematol.

[55] Subramaniam PS, Whye DW, Efimenko E, Chen J, Tosello V, De Keersmaecker K (2012). Targeting nonclassical oncogenes for therapy in T-ALL. Cancer Cell.

[56] Dibirdik I, Langlie MC, Ledbetter JA, Tuel-Ahlgren L, Obuz V, Waddick KG (1991). Engagement of interleukin-7 receptor stimulates tyrosine phosphorylation, phosphoinositide turnover, and clonal proliferation of human T-lineage acute lymphoblastic leukemia cells. Blood.

[57] Medyouf H, Gusscott S, Wang H, Tseng JC, Wai C, Nemirovsky O (2011). High-level IGF1R expression is required for leukemia-initiating cell activity in T-ALL and is supported by Notch signaling. J Exp Med.

[58] Trimarchi T, Bilal E, Ntziachristos P, Fabbri G, Dalla-Favera R, Tsirigos A (2014). Genome-wide mapping and characterization of notch-regulated long noncoding RNAs in acute leukemia. Cell.

[59] Lapidot T, Sirard C, Vormoor J, Murdoch B, Hoang T, Caceres-Cortes J (1994). A cell initiating human acute myeloid leukaemia after transplantation into SCID mice. Nature.

[60] Notta F, Mullighan CG, Wang JC, Poeppl A, Doulatov S, Phillips LA (2011). Evolution of human BCR-ABL1 lymphoblastic leukaemia-initiating cells. Nature.

[61] Guo W, Lasky JL, Chang CJ, Mosessian S, Lewis X, Xiao Y (2008). Multi-genetic events collaboratively contribute to Pten-null leukaemia stem-cell formation. Nature.

[62] Cox CV, Martin HM, Kearns PR, Virgo P, Evely RS, Blair A (2007). Characterization of a progenitor cell population in childhood T-cell acute lymphoblastic leukemia. Blood.

[63] Patel B, Dey A, Castleton AZ, Schwab C, Samuel E, Sivakumaran J (2014). Mouse xenograft modeling of human adult acute lymphoblastic leukemia provides mechanistic insights into adult LIC biology. Blood.

[64] Eppert K, Takenaka K, Lechman ER, Waldron L, Nilsson B, van Galen P (2011). Stem cell gene expression programs influence clinical outcome in human leukemia. Nat Med.

[65] Guo W, Schubbert S, Chen JY, Valamehr B, Mosessian S, Shi H (2011). Suppression of leukemia development caused by PTEN loss. Proc Natl Acad Sci U S A.

[66] Yilmaz OH, Valdez R, Theisen BK, Guo W, Ferguson DO, Wu H (2006). Pten dependence distinguishes haematopoietic stem cells from leukaemia-initiating cells. Nature.

[67] Kalaitzidis D, Sykes SM, Wang Z, Punt N, Tang Y, Ragu C (2012). mTOR complex 1 plays critical roles in hematopoiesis and Pten-loss-evoked leukemogenesis. Cell Stem Cell.

[68] Magee JA, Ikenoue T, Nakada D, Lee JY, Guan KL, Morrison SJ (2012). Temporal changes in PTEN and mTORC2 regulation of hematopoietic stem cell self-renewal and leukemia suppression. Cell Stem Cell.

[69] Hoshii T, Tadokoro Y, Naka K, Ooshio T, Muraguchi T, Sugiyama N (2012). mTORC1 is essential for leukemia propagation but not stem cell self-renewal. J Clin Invest.

[70] Blackburn JS, Liu S, Wilder JL, Dobrinski KP, Lobbardi R, Moore FE (2014). Clonal evolution enhances leukemia-propagating cell frequency in T cell acute lymphoblastic leukemia through Akt/mTORC1 pathway activation. Cancer Cell.

[71] Guo F, Zhang S, Grogg M, Cancelas JA, Varney ME, Starczynowski DT (2013). Mouse gene targeting reveals an essential role of mTOR in hematopoietic stem cell engraftment and hematopoiesis. Haematologica.

[72] Tamburini J, Green AS, Bardet V, Chapuis N, Park S, Willems L (2009). Protein synthesis is resistant to rapamycin and constitutes a promising therapeutic target in acute myeloid leukemia. Blood.

[73] Martel RR, Klicius J, Galet S (1977). Inhibition of the immune response by rapamycin, a new antifungal antibiotic. Can J Physiol Pharmacol.

[74] Xu Q, Thompson JE, Carroll M (2005). mTOR regulates cell survival after etoposide treatment in primary AML cells. Blood.

[75] Zeng Z, dos Sarbassov D, Samudio IJ, Yee KW, Munsell MF, Ellen Jackson C (2007). Rapamycin derivatives reduce mTORC2 signaling and inhibit AKT activation in AML. Blood.

[76] Recher C, Beyne-Rauzy O, Demur C, Chicanne G, Dos Santos C, Mas VM (2005). Antileukemic activity of rapamycin in acute myeloid leukemia. Blood.

[77] Perl AE, Kasner MT, Tsai DE, Vogl DT, Loren AW, Schuster SJ (2009). A phase I study of the mammalian target of rapamycin inhibitor sirolimus and MEC chemotherapy in relapsed and refractory acute myelogenous leukemia. Clin Cancer Res.

[78] Hess G, Herbrecht R, Romaguera J, Verhoef G, Crump M, Gisselbrecht C (2009). Phase III study to evaluate temsirolimus compared with investigator's choice therapy for the treatment of relapsed or refractory mantle cell lymphoma. J Clin Oncol.

[79] Amadori S, Stasi R, Martelli AM, Venditti A, Meloni G, Pane F (2011). Temsirolimus, an mTOR inhibitor, in combination with lower-dose clofarabine as salvage therapy for older patients with acute myeloid leukaemia: results of a phase II GIMEMA study (AML-1107). Br J Haematol.

[80] Perl AE, Kasner MT, Shank D, Luger SM, Carroll M (2011). Single-cell pharmacodynamic monitoring of S6 ribosomal protein phosphorylation in AML blasts during a clinical trial combining the mTOR inhibitor sirolimus and intensive chemotherapy. Clin Cancer Res.

[81] Naval Daver M, Kantarjian HM, Thomas DA, Rytting ME, Farhad R, Nitin J (2013). A phase I/II study of hyper-CVAD plus everolimus in patients with relapsed/refractory acute lymphoblastic leukemia. ASH annual meeting and exposition.

[82] O'Brien S, Thomas D, Ravandi F, Faderl S, Cortes J, Borthakur G (2008). Outcome of adults with acute lymphocytic leukemia after second salvage therapy. Cancer.

[83] Hoshii T, Kasada A, Hatakeyama T, Ohtani M, Tadokoro Y, Naka K (2014). Loss of mTOR complex 1 induces developmental blockage in early T-lymphopoiesis and eradicates T-cell acute lymphoblastic leukemia cells. Proc Natl Acad Sci U S A.

[84] Feldman ME, Apsel B, Uotila A, Loewith R, Knight ZA, Ruggero D (2009). Active-site inhibitors of mTOR target rapamycin-resistant outputs of mTORC1 and mTORC2. PLoS Biol.

[85] Janes MR, Vu C, Mallya S, Shieh MP, Limon JJ, Li LS (2013). Efficacy of the investigational mTOR kinase inhibitor MLN0128/INK128 in models of B-cell acute lymphoblastic leukemia. Leukemia.

[86] Garcia-Martinez JM, Moran J, Clarke RG, Gray A, Cosulich SC, Chresta CM (2009). Ku-0063794 is a specific inhibitor of the mammalian target of rapamycin (mTOR). Biochem J.

[87] Ezell SA, Mayo M, Bihani T, Tepsuporn S, Wang S, Passino M (2014). Synergistic induction of apoptosis by combination of BTK and dual mTORC1/2 inhibitors in diffuse large B cell lymphoma. Oncotarget.

[88] Ashworth RE, Wu J (2014). Mammalian target of rapamycin inhibition in hepatocellular carcinoma. World J Hepatol.

[89] Sampath D, Cortes J, Estrov Z, Du M, Shi Z, Andreeff M (2006). Pharmacodynamics of cytarabine alone and in combination with 7-hydroxystaurosporine (UCN-01) in AML blasts in vitro and during a clinical trial. Blood.

[90] Gojo I, Perl A, Luger S, Baer MR, Norsworthy KJ, Bauer KS (2013). Phase I study of UCN-01 and perifosine in patients with relapsed and refractory acute leukemias and high-risk myelodysplastic syndrome. Invest New Drugs.

[91] Sampath D, Malik A, Plunkett W, Nowak B, Williams B, Burton M (2013). Phase I clinical, pharmacokinetic, and pharmacodynamic study of the Akt-inhibitor triciribine phosphate monohydrate in patients with advanced hematologic malignancies. Leuk Res.

[92] Fruman DA, Cantley LC (2014). Idelalisib–a PI3Kdelta inhibitor for B-cell cancers. N Engl J Med.

[93] Furman RR, Sharman JP, Coutre SE, Cheson BD, Pagel JM, Hillmen P (2014). Idelalisib and rituximab in relapsed chronic lymphocytic leukemia. N Engl J Med.

[94] Gopal AK, Kahl BS, de Vos S, Wagner-Johnston ND, Schuster SJ, Jurczak WJ (2014). PI3Kdelta inhibition by idelalisib in patients with relapsed indolent lymphoma. N Engl J Med.

[95] Hoellenriegel J, Meadows SA, Sivina M, Wierda WG, Kantarjian H, Keating MJ (2011). The phosphoinositide 3'-kinase delta inhibitor, CAL-101, inhibits B-cell receptor signaling and chemokine networks in chronic lymphocytic leukemia. Blood.

[96] Lopez JP, Jimeno A (2014). Idelalisib for the treatment of indolent non-Hodgkin's lymphoma. Drugs Today (Barc).

[97] Nathalie Y, Rosin P, Ekaterina K, Koehrer S, Wang Z, O'Brien S (2013). The PI3K delta inhibitor idelalisib interferes with pre-B cell receptor signaling in acute lymphoblastic leukemia (ALL): a new therapeutic concept. ASH annual meeting and exposition.

[98] Lannutti BJ, Meadows SA, Herman SE, Kashishian A, Steiner B, Johnson AJ (2011). CAL-101, a p110delta selective phosphatidylinositol-3-kinase inhibitor for the treatment of B-cell malignancies, inhibits PI3K signaling and cellular viability. Blood.

[99] Xiaoyan Huang M, Proctor J, Yang Y, Gao X, Zhang W, Huang S (2013). The potent PI3K-δ,γ inhibitor, IPI-145, exhibits preclinical activity in murine and human T-cell acute lymphoblastic leukemia. ASH annual meeting and exposition.

[100] Steelman LS, Pohnert SC, Shelton JG, Franklin RA, Bertrand FE, McCubrey JA (2004). JAK/STAT, Raf/MEK/ERK, PI3K/Akt and BCR-ABL in cell cycle progression and leukemogenesis. Leukemia.

[101] Vachhani P, Bose P, Rahmani M, Grant S (2014). Rational combination of dual PI3K/mTOR blockade and Bcl-2/-xL inhibition in AML. Physiol Genomics.

[102] Will M, Qin AC, Toy W, Yao Z, Rodrik-Outmezguine V, Schneider C (2014). Rapid induction of apoptosis by PI3K inhibitors is dependent upon their transient inhibition of RAS-ERK signaling. Cancer Discov.

[103] Smith GC, Ong WK, Costa JL, Watson M, Cornish J, Grey A (2013). Extended treatment with selective phosphatidylinositol 3-kinase and mTOR inhibitors has effects on metabolism, growth, behaviour and bone strength. FEBS J.

[104] De Buck SS, Jakab A, Boehm M, Bootle D, Juric D, Quadt C (2014). Population pharmacokinetics and pharmacodynamics of BYL719, a phosphoinositide 3-kinase antagonist, in adult patients with advanced solid malignancies. Br J Clin Pharmacol.

[105] Brown JR, Byrd JC, Coutre SE, Benson DM, Flinn IW, Wagner-Johnston ND (2014). Idelalisib, an inhibitor of phosphatidylinositol 3-kinase p110delta, for relapsed/refractory chronic lymphocytic leukemia. Blood.

[106] Kahl BS, Spurgeon SE, Furman RR, Flinn IW, Coutre SE, Brown JR (2014). A phase 1 study of the PI3Kdelta inhibitor idelalisib in patients with relapsed/refractory mantle cell lymphoma (MCL). Blood.

[107] Naval Daver HK, DeBose LK, Jabbour E, Borthakur G, Pemmaraju N, Garcia-Manero G (2014). Buparlisib, a PI3K inhibitor, demonstrates acceptable tolerability and preliminary activity in a phase I/II trial of patients with advanced leukemias. EHA 19.

[108] Lonetti AAC, Spartà AM, Bressanin D, Francesca B, Francesca C, Evangelisti C (2014). Inhibition of class I phosphatidylinositol 3-kinases (pi3ks) isoforms in t-cell acute lymphoblastic leukemia (t-all): which is the best therapeutic strategy?. 19th annual meeting of EHA. Milan, Italy.

[109] Lonetti A, Antunes IL, Chiarini F, Orsini E, Buontempo F, Ricci F (2014). Activity of the pan-class I phosphoinositide 3-kinase inhibitor NVP-BKM120 in T-cell acute lymphoblastic leukemia. Leukemia.

[110] Dail MP, Wong J, Lawrence J, O'Connor D, Nakitandwe J, Chen S-C (2013). Preclinical testing of a PI3K inhibitor in T lineage leukemia: target validation and Notch1/Myc down-regulation in drug resistant clones. ASH annual meeting and exposition.

[111] Wunderle LM, Badura S, Lang F, Wolf A, Schleyer E, Serve H (2013). Safety and efficacy of BEZ235, a dual PI3-kinase/mTOR inhibitor, in adult patients with relapsed or refractory acute leukemia: results of a phase I study. ASH annual meeting and exposition.

[112] Tasian SKM, Li Y, Ryan T, Vincent T, Teachey DT, Loh ML (2013). In vivo efficacy of PI3K pathway signaling inhibition for Philadelphia chromosome-like acute lymphoblastic leukemia. ASH annual meeting and exposition.

[113] Badura S, Tesanovic T, Pfeifer H, Wystub S, Nijmeijer BA, Liebermann M (2013). Differential effects of selective inhibitors targeting the PI3K/AKT/mTOR pathway in acute lymphoblastic leukemia. PLoS One.

[114] Okabe S, Tauchi T, Tanaka Y, Kitahara T, Kimura S, Maekawa T (2014). Efficacy of the dual PI3K and mTOR inhibitor NVP-BEZ235 in combination with nilotinib against BCR-ABL-positive leukemia cells involves the ABL kinase domain mutation. Cancer Biol Ther.

[115] Schult C, Dahlhaus M, Glass A, Fischer K, Lange S, Freund M (2012). The dual kinase inhibitor NVP-BEZ235 in combination with cytotoxic drugs exerts anti-proliferative activity towards acute lymphoblastic leukemia cells. Anticancer Res.

[116] Chiarini F, Fala F, Tazzari PL, Ricci F, Astolfi A, Pession A (2009). Dual inhibition of class IA phosphatidylinositol 3-kinase and mammalian target of rapamycin as a new therapeutic option for T-cell acute lymphoblastic leukemia. Cancer Res.

[117] Shepherd C, Banerjee L, Cheung CW, Mansour MR, Jenkinson S, Gale RE (2013). PI3K/mTOR inhibition upregulates NOTCH-MYC signalling leading to an impaired cytotoxic response. Leukemia.

[118] Serra V, Markman B, Scaltriti M, Eichhorn PJ, Valero V, Guzman M (2008). NVP-BEZ235, a dual PI3K/mTOR inhibitor, prevents PI3K signaling and inhibits the growth of cancer cells with activating PI3K mutations. Cancer Res.

[119] Medvetz D, Priolo C, Henske EP (2015). Therapeutic targeting of cellular metabolism in cells with hyperactive mTORC1: a paradigm shift. Mol Cancer Res.

[120] Bertacchini J, Guida M, Accordi B, Mediani L, Martelli AM, Barozzi P (2014). Feedbacks and adaptive capabilities of the PI3K/Akt/mTOR axis in acute myeloid leukemia revealed by pathway selective inhibition and phosphoproteome analysis. Leukemia.

[121] Wang JM, Chao JR, Chen W, Kuo ML, Yen JJ, Yang-Yen HF (1999). The antiapoptotic gene mcl-1 is up-regulated by the phosphatidylinositol 3-kinase/Akt signaling pathway through a transcription factor complex containing CREB. Mol Cell Biol.

[122] Pugazhenthi S, Nesterova A, Sable C, Heidenreich KA, Boxer LM, Heasley LE (2000). Akt/protein kinase B up-regulates Bcl-2 expression through cAMP-response element-binding protein. J Biol Chem.

[123] Rahmani M, Aust MM, Attkisson E, Williams DC, Ferreira-Gonzalez A, Grant S (2013). Dual inhibition of Bcl-2 and Bcl-xL strikingly enhances PI3K inhibition-induced apoptosis in human myeloid leukemia cells through a GSK3- and Bim-dependent mechanism. Cancer Res.

[124] Sos ML, Fischer S, Ullrich R, Peifer M, Heuckmann JM, Koker M (2009). Identifying genotype-dependent efficacy of single and combined PI3K- and MAPK-pathway inhibition in cancer. Proc Natl Acad Sci U S A.

[125] Fan AC, O'Rourke JJ, Praharaj DR, Felsher DW (2013). Real-time nanoscale proteomic analysis of the novel multi-kinase pathway inhibitor rigosertib to measure the response to treatment of cancer. Expert Opin Investig Drugs.

[126] Seetharam M, Fan AC, Tran M, Xu L, Renschler JP, Felsher DW (2012). Treatment of higher risk myelodysplastic syndrome patients unresponsive to hypomethylating agents with ON 01910.Na. Leuk Res.

[127] Garcia-Manero GM, Fenaux P, Al-Kali A, Baer MR, Sekeres MA, Roboz GJ (2014). Overall survival and subgroup analysis from a randomized phase III study of intravenous rigosertib versus best supportive care (BSC) in patients (pts) with higher-risk myelodysplastic syndrome (HR-MDS) after failure of hypomethylating agents (HMAs). ASH annual meeting and exposition.

[128] Ali K, Soond DR, Pineiro R, Hagemann T, Pearce W, Lim EL, et al. Inactivation of PI(3)K p110delta breaks regulatory T-cell-mediated immune tolerance to cancer. Nature;509(7505):407–11. 10.1038/nature13444 PMC4501086 24919154

[129] Maecker H (2014). Harnessing the power of the immune system to treat cancer.

[130] Dreyling MMF, Bron D, Bouabdallah K, Vitolo U, Linton K, Van den Neste E (2013). Preliminary results of a phase II study of single agent bay 80–6946, a novel PI3K inhibitor, in patients with relapsed/refractory, indolent or aggressive lymphoma. ASH annual meeting and exposition.

[131] Roschewski M, Farooqui M, Aue G, Wilhelm F, Wiestner A (2013). Phase I study of ON 01910.Na (Rigosertib), a multikinase PI3K inhibitor in relapsed/refractory B-cell malignancies. Leukemia.

[132] Komrokji RS, Raza A, Lancet JE, Ren C, Taft D, Maniar M (2013). Phase I clinical trial of oral rigosertib in patients with myelodysplastic syndromes. Br J Haematol.

[133] Zeng Z, Samudio IJ, Zhang W, Estrov Z, Pelicano H, Harris D (2006). Simultaneous inhibition of PDK1/AKT and Fms-like tyrosine kinase 3 signaling by a small-molecule KP372-1 induces mitochondrial dysfunction and apoptosis in acute myelogenous leukemia. Cancer Res.

[134] Friedman DR, Lanasa MC, Davis PH, Allgood SD, Matta KM, Brander DM (2014). Perifosine treatment in chronic lymphocytic leukemia: results of a phase II clinical trial and in vitro studies. Leuk Lymphoma.

[135] Dumble M, Crouthamel MC, Zhang SY, Schaber M, Levy D, Robell K (2014). Discovery of novel AKT inhibitors with enhanced anti-tumor effects in combination with the MEK inhibitor. PLoS One.

[136] Levy DS, Kahana JA, Kumar R (2009). AKT inhibitor, GSK690693, induces growth inhibition and apoptosis in acute lymphoblastic leukemia cell lines. Blood.

